# Spatiotemporal Ecologic Analysis of COVID-19 Vaccination Coverage and Outcomes, Oklahoma, USA, February 2020–December 2021

**DOI:** 10.3201/eid3011.231582

**Published:** 2024-11

**Authors:** Kai Ding, Ozair H. Naqvi, R. Jackson Seeberger, Dale W. Bratzler, Aaron M. Wendelboe

**Affiliations:** University of Oklahoma Health Sciences, Oklahoma City, Oklahoma, USA

**Keywords:** COVID-19, SARS-COV-2, viruses, respiratory infections, zoonoses, cumulative death rate, cumulative hospitalization rate, spatiotemporal analysis, vaccination coverage, United States

## Abstract

Data on COVID-19 cases, deaths, hospitalizations, and vaccinations in Oklahoma, USA, have not been systematically described. The relationship between vaccination and COVID-19–related outcomes over time has not been investigated. We graphically described data collected during February 2020–December 2021 and conducted spatiotemporal modeling of monthly increases in COVID-19 cumulative death and hospitalization rates, adjusting for cumulative case rate, to explore the relationship. A 1 percentage point increase (absolute change) in the cumulative vaccination rate was associated with a 6.3% (95% CI 1.4%–10.9%) relative decrease in death outcome during April–June 2021, and a 1.9% (95% CI 1.1%–2.6%) relative decrease in death outcome and 1.1% (95% CI 0.5%–1.7%) relative decrease in hospitalization outcome during July–December 2021; the effect on hospitalizations was driven largely by data from urban counties. Our findings from Oklahoma suggest that increasing cumulative vaccination rates might reduce the increase in cumulative death and hospitalization rates from COVID-19.

COVID-19 dramatically increased severe outcomes in the United States, based on >5 million hospitalizations and >1 million deaths being reported as of late 2022 (*1*). In Oklahoma (Figure 1), a state in the south-central United States, the pandemic resulted in >101,000 hospitalizations and >14,000 deaths during that period (*1*). On the basis of provisional mortality data from the Centers for Disease Control and Prevention (CDC), COVID-19 was the third leading cause of death in the United States in both 2020 and 2021 (*2*,*3*). Furthermore, COVID-19 led to decreases in US life expectancy from 77.3 years in 2020 to 76.1 years in 2021 (*4*,*5*). In late 2020, the US Food and Drug Administration provided emergency use authorization for 2 separate COVID-19 vaccines, developed by Pfizer-BioNTech (https://www.pfizer.com) and Moderna (https://www.modernatx.com) pharmaceutical companies, followed promptly by recommendations from the Advisory Committee on Immunization Practices for prioritization and use of the vaccines (*6*,*7*). By April 2021, COVID-19 vaccines were available in Oklahoma for all persons >16 years of age (*8*). At that point, ≈5,000 deaths involving COVID-19 had occurred in the state, making the need for further medical interventions critical to preventing further loss of life (*1*). The resulting vaccination campaign led to declines in rates of COVID-19 incidence, emergency department visits, hospitalizations, and deaths across the nation (*9*). 

**Figure 1:**
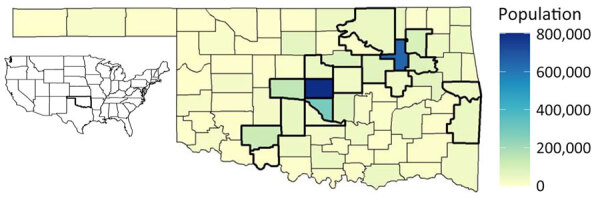
Geography of Oklahoma, USA, and population by county (range 2,137–797,434 residents). Counties with thick boundary lines are metropolitan. The 2 most populous are Oklahoma County (population 797,434) and Tulsa County (population 651,552). Inset map shows location of Oklahoma in the mainland United States.

However, implementation of preventive measures and severe COVID-19 outcomes were uneven depending on where persons lived (*10*–*12*). Despite the disease’s severity, many persons living in the United States have been skeptical about their risk of experiencing a COVID-19–related hospitalization or death and even more skeptical about receiving a COVID-19 vaccine to reduce such risks (*13*–*15*). Those views have been disproportionately shared by persons living in rural areas, based on the unsupported belief that COVID-19 poses a greater risk in urban settings (*15*–*17*). Current evidence indicates that rural residents are less likely to vaccinate against COVID-19 than are urban residents (*18*–*20*). For persons less skeptical of vaccination, the risk factors associated with adverse COVID-19 outcomes, such as older persons living in multigenerational households or lower socioeconomic status among residents of sparsely populated regions, should encourage COVID-19 vaccine uptake (*19*,*21**,**22*). Moreover, analyses of cumulative death rates from COVID-19 have pointed to disproportionate burdens borne by residents of rural compared with urban counties (*23*,*24*). 

Similar disparities have been reported in terms of COVID-19 vaccination coverage, which is lower in rural than urban counties in Oklahoma (*25*), including in its 2 large metropolitan counties. Vaccination coverage even varied among counties by a wide margin, from 43% of persons receiving >1 dose in rural Cimarron County (county seat Boise City) to 88.3% in mostly urban Oklahoma County (county seat Oklahoma City) as of December 2021 (*26*). Other studies have also described disparities between urban and rural counties in COVID-19 vaccination coverage; that coverage gap more than doubled from April 2021 through January 2022 (*25*,*27*). Although national studies have linked counties on the fringes of large metropolitan areas and nonmetropolitan counties to greater COVID-19 disparities (*28*), further research is needed to characterize that relationship between county metropolitan status and vaccination coverage on the state level to evaluate how local public health interventions to increase vaccination coverage can be improved. 

Spatiotemporal epidemiology can be used to integrate the investigation of health outcomes across geography and time (*29*,*30*). Prior studies have taken county of residence and underlying medical conditions into consideration when evaluating the spread of COVID-19 across communities over time (*31*–*33*); however, the time-varying effect of vaccination on both death and hospitalization related to COVID-19 has not been thoroughly explored.

We used county-level COVID-19 data from Oklahoma to conduct a spatiotemporal ecologic study with 2 objectives: to describe the distribution of COVID-19–related cases, deaths, hospitalizations, and vaccinations over time and to investigate the correlation between COVID-19 cumulative death and hospitalization rates and vaccination coverage. Furthermore, we assessed whether the correlation varied between urban and rural counties. The University of Oklahoma Health Sciences institutional review board determined this study (review no. 17463) did not meet criteria for human subjects research. 

## Methods 

### Data Sources 

We obtained county-level cumulative vaccination rates and individual-level data on COVID-19 cases and outcomes (death and hospitalization) from the CDC National Notifiable Disease Surveillance System (NNDSS) COVID-19 Case Surveillance Restricted Access Dataset (*34*). The dataset included deidentified individual-level data on confirmed and probable COVID-19 cases, hospitalizations, and deaths reported from local and state public health jurisdictions (*35*). We accessed data for the excess death plot from the CDC National Center for Health Statistics, modified it to reflect the February 2020–December 2021 study period, and accessed county-level vaccination data from the CDC COVID Data Tracker, which included county-level population data (*1*,*36*). 

### Measures

The primary exposure was the cumulative vaccination rate, which we defined as the percentage of the county population that had completed the 2-dose series by a given date. We included both probable and laboratory-confirmed COVID-19 cases in our analyses. Because of an issue involving incomplete reporting of death events, we restricted analyses to data collected through December 4, 2021. We defined an absolute cumulative event (COVID-19 case, death, or hospitalization) rate as the county’s cumulative event count on a specific date normalized to 100,000 residents. For spatiotemporal modeling, we calculated the increases in the cumulative rate of COVID-19 death and hospitalization outcomes over a time interval by subtracting the county’s absolute cumulative rate on the day before the start of the time interval from that rate at the end of the time interval. We defined counties as urban if designated metropolitan according to the CDC National Center for Health Statistics urban/rural classification scheme for counties (*37*). 

### Statistical Analysis

**Figure 2:**
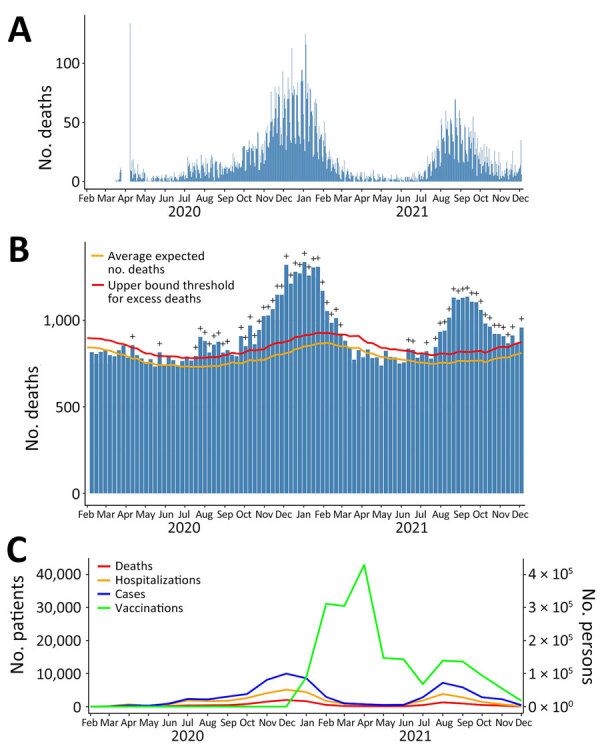
Distribution of COVID-19 deaths (A), all-cause and excess mortality (B), and COVID-19 cases, hospitalization, deaths, and rates of vaccination against COVID-19 (C) in analysis of COVID-19 vaccination coverage and outcomes, Oklahoma, USA, February 2020–December 2021. The spike in the number of deaths on April 8, 2020, was caused by the delay in death reporting early in the pandemic. The excess mortality plot was accessed from the Centers for Disease Control and Prevention National Center for Health Statistics and modified to reflect the study period February 2020–December 2021. Plus (+) symbol indicates observed count above threshold (defined as the upper bound of the 95% prediction interval of the expected number of deaths). In panel C, scales for the y-axes differ substantially to underscore patterns but do not permit direct comparisons. Data sources: https://covid.cdc.gov/covid-data-tracker; https://data.cdc.gov/Case-Surveillance/COVID-19-Case-Surveillance-Restricted-Access-Detai/mbd7-r32t

We determined county-level cumulative vaccination, death, and hospitalization rates at selected time points in spatial plots. We computed Pearson correlation coefficients and 95% CIs between cumulative vaccination rates and cumulative rates of outcomes weighted by county population size at selected time points. We also used scatter plots with weighted least-squares lines to visualize the relationship between vaccination coverage and the absolute cumulative outcome rates. On the basis of epidemic curve of COVID-19–related deaths (Figure 2, panel A), we studied the relationship between cumulative vaccination rates and COVID-19–related outcomes during 3 intervals: January 1–March 31, 2021; April 1–June 30, 2021; and July 1–December 4, 2021. Within each time interval, we created a scatter plot of the averaged cumulative vaccination rate, calculated as the average of the cumulative vaccination rates at the start and the end dates of the interval, compared with the increase in the cumulative death or hospitalization rate during the same interval. 

We based spatiotemporal analysis (Appendix) on the generalized additive model, including a linear term in averaged cumulative vaccination rate and a nonparametric function of the spatial location and time. Specifically, for each interval, we modeled county-level monthly data on averaged cumulative vaccination rates and the increase in cumulative death or hospitalization rates by mixed-effects quasi-Poisson regression using the function GAM in the R package MGCV (The R Project for Statistical Computing, https://www.r-project.com), where the spatiotemporal data structure was captured through a tensor-product of spline-based spatial and temporal basis functions and the tuning parameters in the number of knots were selected based on the generalized cross-validation score (*30*). All models adjusted for the monthly cumulative COVID-19 case rate (averaged between the first and last days of each month) as a potential confounder. In those models, we treated the averaged cumulative vaccination and cumulative COVID-19 case rates as fixed effects and used the tensor-product terms with random coefficients (random effects) to model the spatial and temporal correlation in the data. We chose the quasi-Poisson model, in which we used the log link function and logarithm of the county population as the offset, to account for overdispersion in the data; all dispersion parameter estimates were >1. We also investigated the interaction between averaged cumulative vaccination rate and county metropolitan status and presented stratified results where warranted. We reported both point estimates and 95% CIs. Statistical significance was reached if the 2-sided p value was <0.05. We performed all analyses using R software version 4.2.1 (The R Project for Statistical Computing https://cran.r-project.org/bin/windows/base/old/4.2.1). 

## Results

### COVID-19–Related Cases, Deaths, Hospitalizations, and Vaccinations over Time

As of December 4, 2021, the data cutoff date for our study, 663,350 reported cases, 11,962 reported provisional deaths, and 38,232 hospitalizations had been attributed to COVID-19 across all 77 counties in Oklahoma. Of those totals, 308,694 (46.5%) cases, 5,914 (49.4%) deaths, and 18,760 (49.1%) hospitalizations were reported by December 31, 2020, before COVID-19 vaccines were sufficiently available to have a meaningful effect on reported cases. We plotted the epidemic curve for COVID-19–related deaths in Oklahoma (Figure 2, panel A). Excess mortality from all causes followed the same trend over time as COVID-19 deaths (Figure 2, panels A, B). We also plotted epidemic curves by month for COVID-19 cases, deaths, patients ever hospitalized, and prime doses of COVID-19 vaccines (Figure 2, panel C). COVID-19–related hospitalizations and deaths followed similar time trends as cases, and peaks were associated with multiple SARS-CoV-2 variants in January 2021 and the Delta variant in August 2021. There was an initial demand for COVID-19 vaccines when they were first made available; peak distribution occurred in March 2021. Demand has largely decreased over time, except for a temporary increase in demand during the August 2021 surge in cases caused by the Delta variant. 

### County-Level COVID-19–Related Death and Vaccination

**Figure 3:**
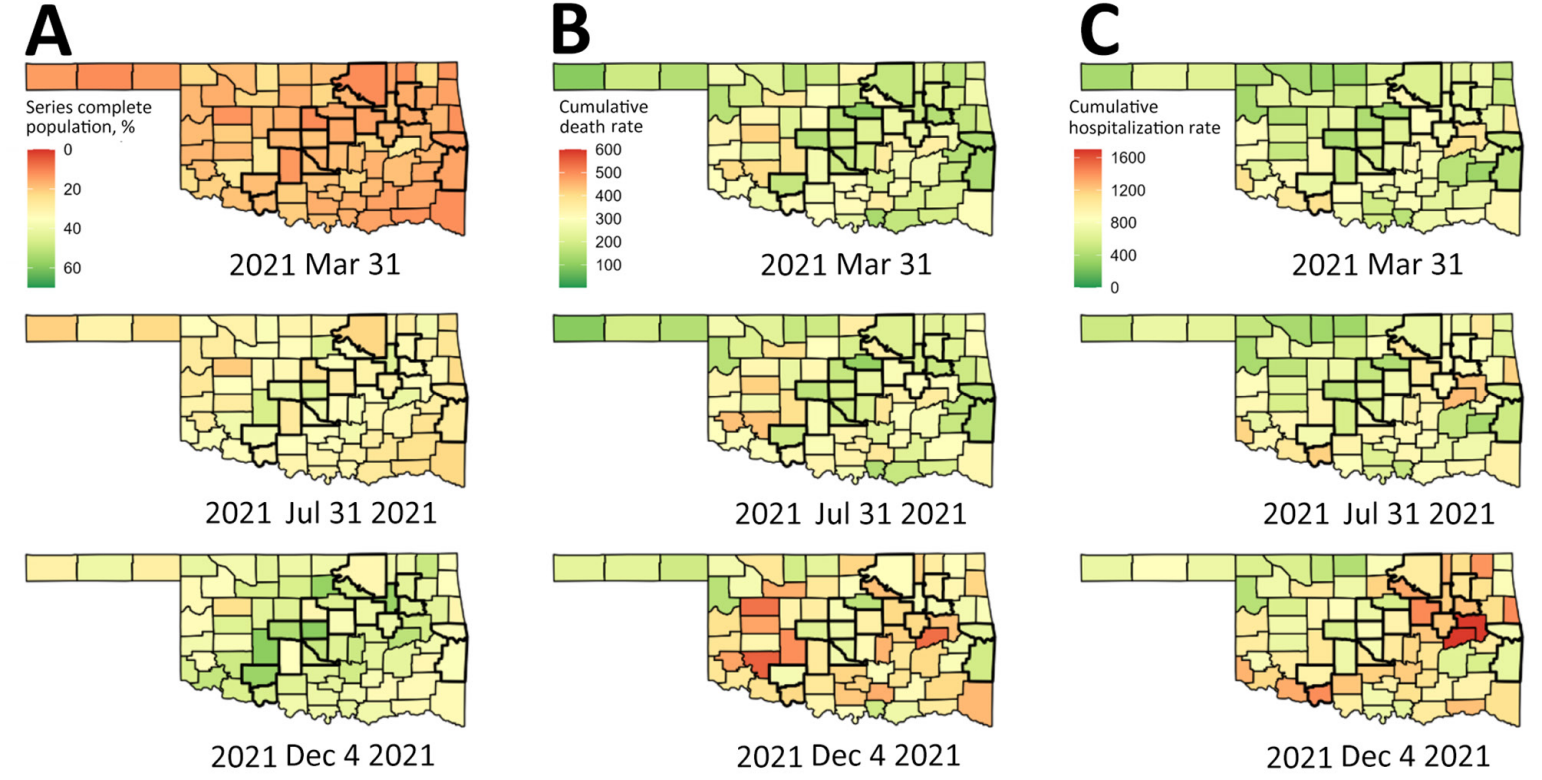
Spatial plots of county-level cumulative vaccination rates and cumulative death and hospitalization rates per 100,000 residents at selected time points in analysis of COVID-19 vaccination coverage and outcomes, Oklahoma, USA, February 2020–December 2021. A) Vaccination rates; B) death rates; C) hospitalization rates. Counties with thick boundary lines are metropolitan.

**Figure 4:**
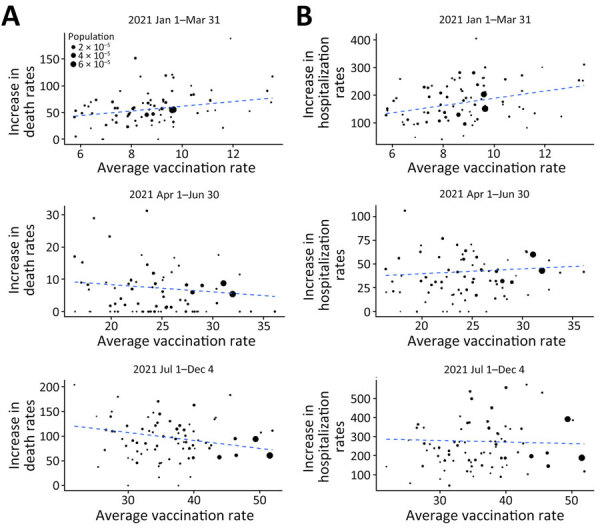
Scatter plot of county-level population percentage of complete vaccination series (averaged between the start and the end dates) versus increase in cumulative death rates (A) and cumulative hospitalization rates (B) per 100,000 residents for selected time intervals in analysis of COVID-19 vaccination coverage and outcomes, Oklahoma, USA, February 2020–December 2021. The dashed line is the weighted (by county population size) least-squares line. The 2 largest solid dots in the plot correspond to the 2 largest counties (i.e., Oklahoma and Tulsa) in Oklahoma.

We used spatial plots to visualize county-level cumulative vaccination and cumulative death rates at selected time points (Figure 3). Among the 77 counties in Oklahoma, by December 4, 2021, cumulative vaccination rates were 24.1%–59.1% (weighted average 48.8%); cumulative death rates were 155.5–551.2 (weighted average 302.3) deaths/100,000 residents. We also calculated weighted Pearson correlation coefficients between cumulative vaccination and cumulative death rates at selected time points (Table 1). Except for the first time point, at which we observed a positive correlation, cumulative vaccination and cumulative death rates were negatively correlated, and the magnitudes of association were moderate (Appendix Figure 1). We also illustrated county-level averaged cumulative vaccination rates versus increases in cumulative death rates per 100,000 residents for selected time intervals (Figure 4, panel A). Again, except during the January 1–March 31, 2021 time period, averaged cumulative vaccination rates and increases in cumulative death rates were negatively associated (i.e., for April 1–June 30, 2021 and July 1–December 4, 2021). 

**Table 1:** Pearson correlations and 95% CIs between cumulative vaccination rate and COVID-related outcomes at selected time points in analysis of COVID-19 vaccination coverage and outcomes, Oklahoma, USA, February 2020–December 2021*

Date	Cumulative death rate (crude 95% CIs)	Cumulative hospitalization rate (crude 95% CIs)
2021 Mar 31	0.182 (0.135–0.223)	0.250 (0.204–0.290)
2021 Jul 31	–0.319 (–0.362 to –0.281)	–0.037 (–0.082 to 0.009)
2021 Dec 4	–0.391 (–0.432 to –0.355)	–0.013 (–0.059 to 0.033)

*95% Cis weighted by county population size; crude 95% CIs were reported.

Through modeling monthly data on averaged cumulative vaccination rates and increases in cumulative death rates (death outcome), a 1 percentage point increase (absolute change) in cumulative vaccination rate was associated with a decrease of 6.3% (95% CI 1.4%–10.9%; p = 0.014) (relative change) in the death outcome for the April–June 2021 time interval and a decrease of 1.9% (95% CI 1.1%–2.6%; p<0.0001) for the July–December 2021 time interval (Table 2); however, we found no association for the January–March 2021 time interval. The interaction between cumulative vaccination rates and metropolitan status of the county was not significant (p = 0.282 for January–March 2021, 0.144 for April–June 2021 and 0.125 for July–December 2021), suggesting that the association between cumulative vaccination rates and the death outcome also did not change according to county metropolitan status. We obtained similar results from sensitivity analyses using the cumulative vaccination rate on the first day of each month, and by including or excluding December 2021 (results not shown). 

**Table 2:** Association between population percentage of complete vaccination series and increases in cumulative death rates and cumulative hospitalization rates, respectively, for selected time intervals in analysis of COVID-19 vaccination coverage and outcomes, Oklahoma, USA, February 2020–December 2021*

Time interval	Death			Hospitalization	
	Relative decrease for 1 percentage point increase in vaccination (95% CI)	p value		Relative decrease for 1 percentage point increase in vaccination (95% CI)	p value
Jan–Mar 2021	–4.4% (–10.5% to 1.4%)	0.130		–7.4% (–11.0% to –3.8%)	<0.0001
Apr–Jun 2021	6.3% (1.4%–10.9%)	0.014		1.1% (–0.7%–2.8%)	0.231
Jul–Dec 2021	1.9% (1.1%–2.6%)	<0.0001		1.1% (0.5%–1.7%)	0.001

*Monthly data were modeled with (log-link) quasi-Poisson regression using the GAM function in R package “MGCV.” All models adjusted for averaged cumulative case rate as potential confounder. †Sensitivity analysis showed similar results when December 2021 data were excluded or using cumulative vaccination rate on the first day of each month

### County-Level COVID-19–Related Hospitalization and Vaccination

We used spatial plots to visualize county-level cumulative vaccination rates and cumulative hospitalization rates at selected time points (Figure 3). Among the 77 counties in Oklahoma, by December 4, 2021, cumulative hospitalization rates were 415.4–1,678.9 (weighted average 966.2)/100,000 residents. We found a positive association between cumulative vaccination and cumulative hospitalization rates for the first time point, but the correlation coefficient was negative for the second and the third time points; however, the magnitude of the negative associations was relatively small (Table 1). We generated scatter plots to illustrate those associations (Appendix Figure 2). We also generated scatter plots of county-level averaged cumulative vaccination rates versus increases in cumulative hospitalization rates per 100,000 residents for selected time intervals (Figure 4, panel B). Again, descriptively, we found a positive association for the January 1–March 31, 2021 time period but observed little association for the April 1–June 30, 2021 and July 1–December 4, 2021 time periods. 

Through modeling monthly data on averaged cumulative vaccination rates and increases in cumulative hospitalization rates (hospitalization outcome), we found a significant positive association for the time interval January–March 2021, but found no significant association for the time interval April–June 2021 (Table 2). For the time interval July–December 2021, there was a negative association, but the magnitude of association was relatively small; a 1 percentage point increase (absolute change) in cumulative vaccination rate was associated with a decrease of 1.1% (95% CI 0.5%–1.7%; p = 0.001) (relative change) in the hospitalization outcome. The p values for the interaction between cumulative vaccination rates and county metropolitan status were 0.023 for January–March 2021, 0.173 for April–June 2021, and 0.031 for July–December 2021. Those values suggest that the association between cumulative vaccination rates and the hospitalization outcome differed according to county metropolitan status for the first and the third time periods but not for the second. The overall negative association for the time interval July–December 2021 was largely driven by data from metropolitan counties (Table 3). We obtained similar results from sensitivity analyses using the cumulative vaccination rate on the first day of each month and by including or excluding December 2021 (results not shown). 

**Table 3:** Association between population percentage of complete vaccination series and the increases in cumulative hospitalization rate stratified by county metropolitan status, for selected time intervals in analysis of COVID-19 vaccination coverage and outcomes, Oklahoma, USA, February 2020–December 2021*

Time interval	Metropolitan counties			Nonmetropolitan counties	
	Relative decrease for 1 percentage point increase in vaccination (95% CI)	p value		Relative decrease for 1 percentage point increase in vaccination (95% CI)	p value
Jan–Mar 2021	–11.7% (–17.9% to –5.9%)	0.0003		–5.7% (–10.5% to –1.1%)	0.017
Apr–Jun 2021	–0.2% (–2.2% to 1.7%)	0.816		–0.9% (–4.1% to 2.2%)	0.575
Jul–Dec 2021	1.1% (0.2%–1.9%)	0.016		–2.3% (–3.8% to –0.9%)	0.001

*Monthly data were modeled with (log-link) quasi-Poisson regression using the GAM function in R package “MGCV.” All models adjusted for averaged cumulative case rate as potential confounder

## Discussion 

Using county-level data, we conducted a spatiotemporal ecologic analysis to investigate the relationship between both COVID-19–related deaths and hospitalizations and COVID-19 vaccination coverage in the state of Oklahoma, USA. Overall, the findings from this study describe how severe COVID-19–related outcomes changed in Oklahoma over time and based on county urban/rural status. Debate about the accuracy of attributing the correct cause of death to patients diagnosed with COVID-19 has occurred; some persons have been concerned that COVID-19–related deaths were being overreported (*36*) and others concerned those deaths were being underreported (*38*). The time trend of excess deaths from all causes was similar to the epidemic curve of COVID-19–related deaths in Oklahoma, which supports COVID-19 as a cause of excess mortality. 

We found a negative correlation between vaccination coverage and COVID-19–related mortality during April–June 2021 and July–December 2021, meaning that higher vaccination coverage was associated with lower increases in cumulative mortality rates during those time periods. The significant association between cumulative vaccination and cumulative death rates was largely driven by data from Oklahoma and Tulsa Counties, the 2 largest counties in Oklahoma (Figure 4, panel A; Appendix Figure 1). The strength of the association was stronger during April–June 2021 than July–December 2021, indicating a possible waning effect for vaccines in protection from death. For COVID-19–related hospitalizations, we did not find a negative correlation with vaccination coverage until July–December 2021; in addition, the magnitude of association was weaker than for COVID-19–related deaths. We also found a positive association between cumulative vaccination rates and outcomes during the early time periods of our study (through March 31, 2021). However, vaccination coverage was low across all counties during the first few months after vaccines first became available. Although Oklahoma implemented rapid initial rollout of COVID-19 vaccines, a time lag would be expected between when vaccinations first became available and reached sufficient population immunity to observe a protective effect. 

Our findings were consistent with documented national spatial and temporal progression of COVID-19 up through September 2021 (*39*). In addition, another analysis (*40*) underscored the association of spatial vaccination heterogeneity with intensified COVID-19 surges, particularly in rural counties, which constitute most areas with low vaccination rates. Those studies emphasize the pivotal role of COVID-19 vaccination coverage in mitigating effects of the pandemic in urban and rural settings (*39*,*40*). Our study offers granular, state-level insight into that relationship in Oklahoma, elucidating the nuanced relationship between vaccination coverage and severe COVID-19 outcomes in urban versus rural contexts. 

The protective benefit of COVID-19 vaccines has been reported at both the population and individual levels on the basis of data from clinical trials and observational studies (*41*–*48*). Despite that evidence, resistance to uptake of COVID-19 vaccine persists. Through experience as healthcare providers participating in the public health response to the pandemic, we have heard anecdotal accounts of persons from rural counties expressing a belief that risk for COVID-19 infection is lower among persons who live in rural than in urban settings. The data do not support this belief and instead show similar cumulative case rates between urban and rural counties during March 2020–March 2021. Furthermore, studies have linked rural counties with higher CDC Social Vulnerability Index (SVI) scores, which are linked to locations with higher poverty, crowded housing, and other community attributes associated with adverse health outcomes (*49*,*50*). Many counties in Oklahoma score high in the SVI, and many of those same counties report lower rates of vaccination coverage, similar to associations observed on the national level between high SVI scores and low vaccination rates (*28*). 

Among strengths of this study, we provided a systematic description of COVID-19 cases, deaths, hospitalizations, and vaccination data in the state of Oklahoma during different time periods that roughly correspond with surges in case numbers during the timeframes of the original and Delta variant of SARS-COV-2 virus and the time period between those surges. We also used a mixed-effects model to account for the correlations and spatiotemporal structure in our data when evaluating associations between COVID-19 vaccination coverage and outcomes. 

Among limitations of this study, the data we used for analyses were ecologic and aggregate in nature so that we could not determine if persons infected with COVID-19 or who died from COVID-19 had been vaccinated. Second, the exact dates associated with outcomes were not available. Instead, we defined those dates as the earlier of the clinical date (date of illness onset or specimen collection) or the date the case report was received by CDC. Third, we used cumulative vaccination rate over time in our modeling and therefore could not account for the waning effect of vaccines in our analyses. Fourth, although it was not a primary outcome, the cumulative number of COVID-19 cases was potentially undercounted, and discrepancies between urban and rural counties in COVID-19 testing practices might have existed, which might have affected our findings. Last, although we adjusted for cumulative case rates in our models and conclusions were similar after further adjusting for county-level median age and income (data not shown), potential uncontrolled confounding effects cannot be ruled out. 

In conclusion, we found a moderate correlation between higher COVID-19 vaccination coverage and lower increase in cumulative COVID-19 death rates but a weaker association with COVID-19–related hospitalization. Future studies using individual-level data are needed to gain further insight into vaccine efficacy. This study provides evidence of the demonstrable benefit to both urban and rural populations in Oklahoma getting vaccinated against COVID-19. That evidence could aid public health officials, healthcare providers, and others to communicate through written and visual media the likely benefits population-level immunity vaccination can provide. 

Appendix Additional information from spatiotemporal ecologic analysis of COVID-19 vaccination coverage and outcomes in Oklahoma during 2020–2021.
