# Supplementary material for: Spatiotemporal Ecologic Analysis of COVID-19 Vaccination Coverage and Outcomes, Oklahoma, USA, February 2020–December 2021
Source: Emerg Infect Dis. 2024 Nov;30(11):2333–42. doi: 10.3201/eid3011.231582 (PMC11521187; doi:10.3201/eid3011.231582)
Supplement: Appendix — Additional information from spatiotemporal ecologic analysis of COVID-19 vaccination coverage and outcomes in Oklahoma during 2020–2021. [file 23-1582-Techapp-s1.pdf]

Article DOI: https://doi.org/10.3201/eid3011.231852

EID cannot ensure accessibility for supplementary materials supplied by authors. Readers who have difficulty accessing supplementary content should contact the authors for assistance.

Spatiotemporal Ecologic Analysis of COVID-19 Vaccination Coverage and Outcomes, Oklahoma, USA, February 2020–December 2021

Appendix

We closely follow an approach to analyzing non-Gaussian spatiotemporal data (*1*). Specifically, we assume a generalized additive model (GAM) of the form

$$\log Y(s;t) = \log N + \beta_0 + \beta_1 e(s;t) + \beta_2 c(s;t) + f(s;t) + v(s;t),$$

where s denotes the space (i.e., latitude and longitude of county centroid) and t the time (i.e., calendar month). $Y(s;t)$ is the increase in cumulative counts of deaths/hospitalizations at space s and time t, $\log N$ is the offset term (here N denotes county population), β_0 is an intercept term, β_1 is regression coefficient for vaccination rate $e(s;t)$ and β_2 for cumulative COVID-19 case rate $c(s;t)$; $f(s;t)$ is a nonparametric random smooth function defined below, and $v(s;t)$ is a spatio-temporal random effect. $Y(s;t)$ is assumed to follow the quasi-Poisson distribution with an overdispersion parameter (i.e., ratio of the variance to the mean). To investigate the interaction between vaccination rate and county metropolitan status m, two additional terms ($\beta_3 m$ and $\beta_4 e(s;t)m$) are added to the above model.

A tensor-product (of basis functions) structure is assumed for $f(s;t)$:

$$f(s;t) = \sum_{i=1}^{k_1}\sum_{j=1}^{k_2} \alpha_{ij}\phi_{1i}(s)\phi_{2j}(t),$$

where α_{ij} are unknown random effects, $\phi_{1i}(s)$ is the spline-based spatial basis function and $\phi_{2j}(t)$ the spline-based temporal basis function, k_1 is the knot count (or number of basis functions) for the spatial component and k_2 is the knot count for the temporal component. We use a thin-plate spline basis over space and a cubic regression spline over time, as described

elsewhere (*1*). We select the tuning parameters k_1 and k_2 based on the generalized cross-validation score.

This modeling approach is implemented using the GAM function in the R package MGCV.

Reference

1. Wikle CK, Zammit-Mangion A, Cressie N. Spatio-temporal statistics with R. Boca Raton [FL]: CRC Press; 2019.

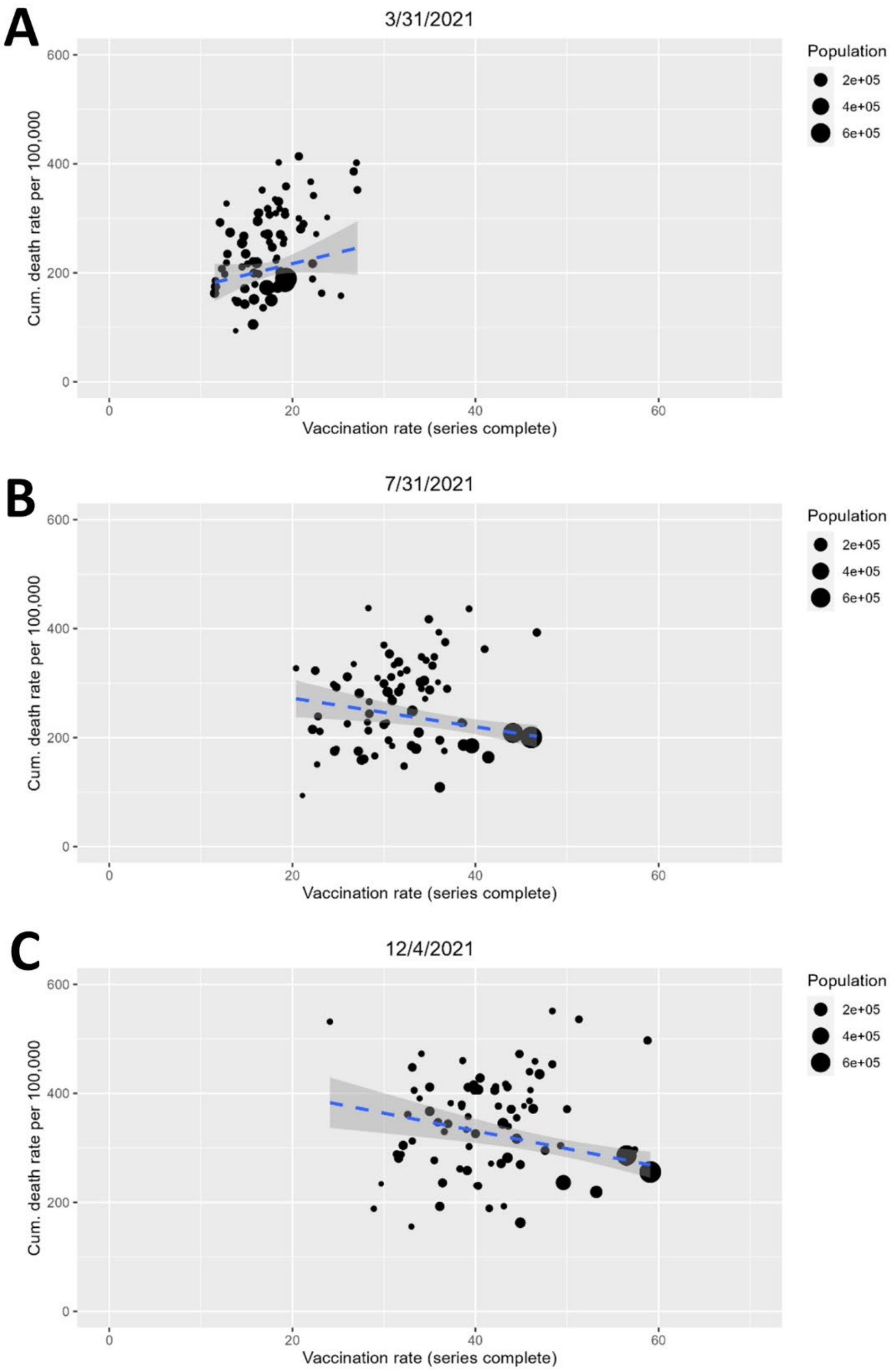

Appendix Figure 1. Scatter plot of county-level population percentage of complete vaccination series and absolute cumulative death rates (per 100,000) at selected time points. The dashed line is the weighted (by county population size) least-squares line. The 2 largest solid dots in the plot correspond to the 2 largest counties in Oklahoma (i.e., Oklahoma and Tulsa).

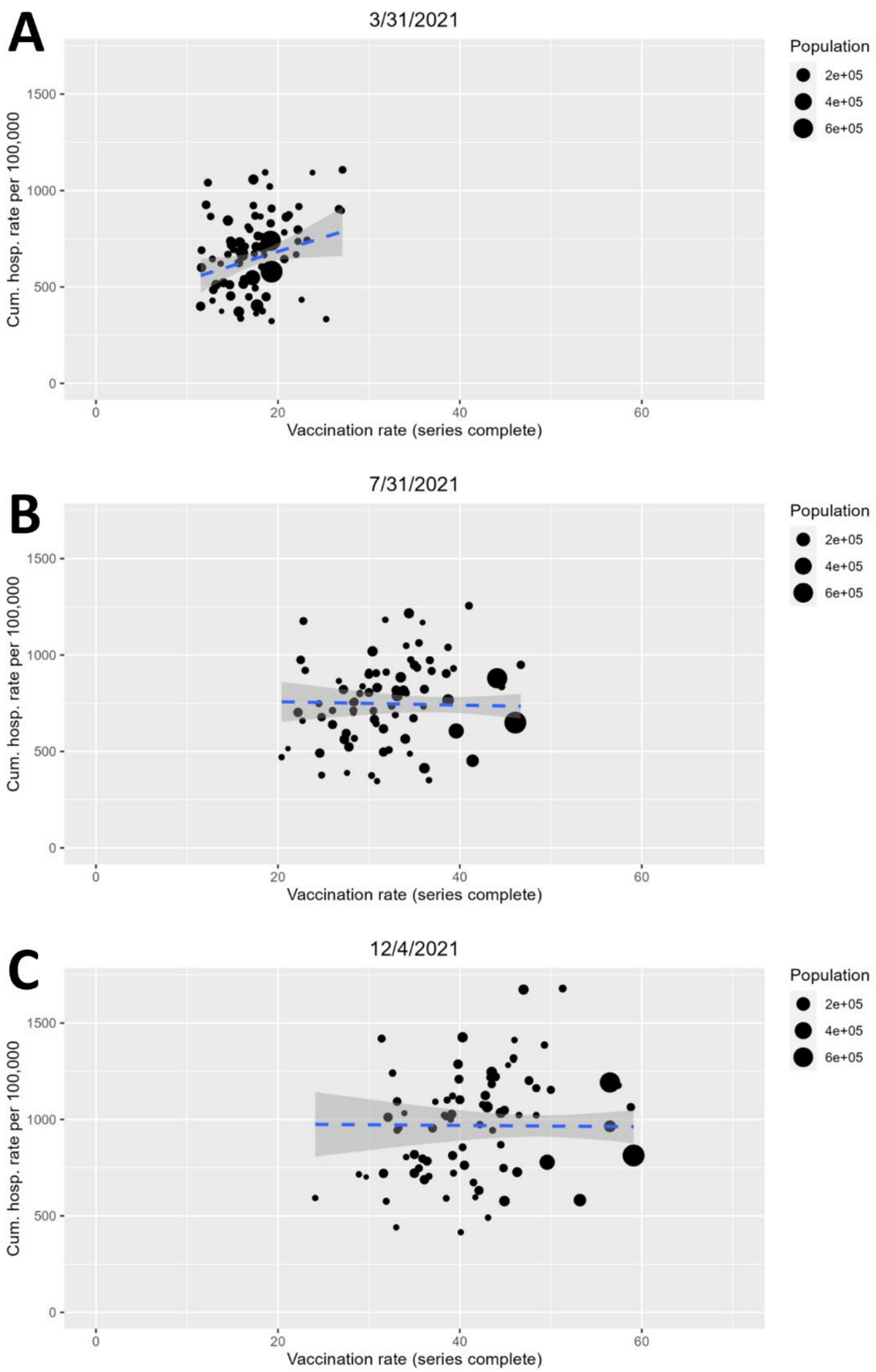

Appendix Figure 2. Scatter plot of county-level population percentage of complete vaccination series and cumulative hospitalization rate (per 100,000) at selected time points. The dashed line is the weighted (by county population size) least-squares line. The 2 largest solid dots in the plot correspond to the 2 largest counties in Oklahoma (i.e., Oklahoma and Tulsa).
